# Testing Procedure for Fatigue Characterization of Steel-CFRP Hybrid Laminate Considering Material Dependent Self-Heating

**DOI:** 10.3390/ma14123394

**Published:** 2021-06-18

**Authors:** Selim Mrzljak, Stefan Schmidt, Andreas Kohl, Daniel Hülsbusch, Joachim Hausmann, Frank Walther

**Affiliations:** 1 Department of Materials Test Engineering (WPT), TU Dortmund University, Baroper Str. 303, D-44227 Dortmund, Germany; andreas.kohl@tu-dortmund.de (A.K.); daniel.huelsbusch@tu-dortmund.de (D.H.); frank.walther@tu-dortmund.de (F.W.); 2 Leibniz-Institut für Verbundwerkstoffe GmbH, Erwin-Schrödinger-Str. 58, D-67663 Kaiserslautern, Germany; stefan.schmidt@ivw.uni-kl.de (S.S.); joachim.hausmann@ivw.uni-kl.de (J.H.)

**Keywords:** fiber metal laminate, thermoplastic, steel, carbon fiber, testing procedure, fatigue behavior, self-heating, residual stress

## Abstract

Combining carbon fiber reinforced polymers (CFRP) with steel offers the potential of utilizing the desired characteristics of both materials, such as specific strength/stiffness and fatigue strength of fiber reinforced polymers (FRP) and impact resistance of metals. Since in such hybrid laminates multiple material layers are combined, a gradual failure is likely that can lead to changes in mechanical properties. A failure of the metal partner leads to an increase in stress on the FRP, which under fatigue load results in increased self-heating of the FRP. Therefore, a suitable testing procedure is required and developed in this study, to enable a reproducible characterization of the mechanical properties under fatigue load. The resulting testing procedure, containing multiple frequency tests as well as load increase and constant amplitude tests, enabled characterization of the fatigue performance while never exceeding a testing induced change in temperature of 4 K. In addition to the development of the testing procedure, an insight into the manufacturing induced residual stresses occurring in such hybrid laminates, which impacts the load-bearing capacity, was established using finite element simulation. The gathered data and knowledge represents a basis for future in-depth investigations in the area of residual stress influence on the performance of hybrid laminates and highlights its importance, since not only the used testing procedure determines the measured fatigue performance.

## 1. Introduction

Hybrid materials such as fiber metal laminates (FML) made of fiber reinforced polymers (FRP) and metals offer the potential of utilizing the desired characteristics of both materials. Especially when considering automotive and aerospace applications, where high performance materials are essential, the specific strength/stiffness and fatigue strength of FRP, as well as impact resistance of metals [1,2,3] is beneficial. Furthermore, the incorporation of a metal constituent can increase the materials poor electrical conductivity potentially enabling additional functionalities such as electrical shielding, grounding, or structural health monitoring [4]. Carbon fiber reinforced polymer (CFRP), being one of the few electrically conductive FRP, represents the superior FRP considering mechanical performance and has already been combined successfully with metals like steel [5], aluminum [6,7,8], or magnesium [9,10] to form hybrid laminates.

However, due to different coefficients of thermal expansion (CTE), thermal residual stresses (TRS) are induced in the manufacturing process while cooling down from processing temperature. Due to the low CTE of FRP in fiber direction compared to metal, tensile TRS occur in the metal constituent [11,12]. Hence, compressive TRS are present in the FRP constituent (in fiber direction). Due to the superposition of TRS, the risk of FRP buckling is increased and the apparent tensile and fatigue strength of the metal is reduced. The latter is due to accelerated crack initiation and propagation by superimposed tensile TRS under cyclic loading [13,14].

A further crucial aspect when considering hybrid laminates for applications under cyclic loading is self-heating, which mainly influences the mechanical properties of the polymer component. This is due to the viscoelastic material behavior of polymers, caused by material damping associated with dissipation of energy [15,16]. The intensity of self-heating is load- as well as frequency-dependent [17,18,19], since the dissipated energy increases with both and therefore the temperature of the material. A simple reduction in frequency to reduce the self-heating is not always sufficient considering the mechanical properties of the polymer [15], since at too low frequencies ratcheting outweighs the benefits of decreased self-heating, reducing ultimately fatigue lifetime. Using the proposed frequency adjustment method by Hülsbusch et al. [15], which considers an energy-based approach and was exemplified on polyamide 6, the testing duration can be kept short while limiting self-heating to a certain temperature. Since hybrid laminates contain multiple materials stacked as layers, partial failure of single layers can occur, leading to damaging of the laminate integrity and therefore changes in load distribution. In case of metal layer failure, while the FRP still withstands the load, increased stress and thereby self-heating results. Therefore, a further factor, the load distribution after partial failure needs to be considered to limit the extend of self-heating.

In this study, a testing procedure for hybrid laminates is developed, which takes material dependent self-heating into account. Profound frequency adjustment depending on the applied stress is carried out, leading to a reproducible testing procedure for the characterization of hybrid laminate fatigue behavior while eliminating the temperature influence. For this purpose, a hybrid laminate consisting of austenitic steel and carbon fiber reinforced polyamide 6 layers was used and successfully characterized using the developed procedure. Accompanying to the fatigue tests electrical resistance measurement and digital image correlation were used to conclude on events of material degradation and damage propagation with regard to evaluation of the fatigue performance and its usability for, e.g., structural health monitoring. In addition, the manufacturing process induced TRS, which impact the load-bearing capacity, as shown by quasistatic tensile tests, were determined using finite element simulation. The results highlight the presence of TRS and therefore the need of considering TRS for future investigations, since not only the used testing procedure determines the measured fatigue performance of a hybrid laminate.

## 2. Materials and Methods

### 2.1. Materials and Manufacturing

**Figure 1:**
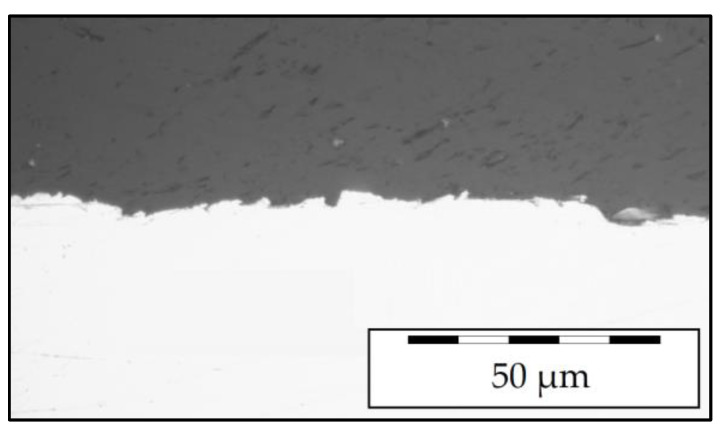
Micrograph of the polymer steel interface with well infiltrated rough metal surface.

Laminate plates were manufactured using unidirectional carbon fiber reinforced polyamide 6 (CFRP) tape (Cetex TC910, Tencate, The Netherlands) with a thickness of 0.15 mm. For the metal part, an austenitic steel sheet (X10CrNi18-8; 1.4310, AISI 301) with a thickness of 0.3 mm was used, which inhibits corrosion at the FRP-metal interface. This combination of CFRP and steel form a generic model material, without a specific industrial application but for the special purpose of developing a testing procedure. To improve the adhesion between the materials through surface treatment of the metal layer [20,21,22,23], the steel sheets were grid blasted with alumina. In addition, polymer foil (PA6, thickness 0.05 mm) was placed between composite and metal layers to improve adhesion, as the excess polymer fills the roughness of the blasted metal surface (Figure 1). The unidirectional hybrid laminates were manufactured in a metal to FRP configuration of 1 to 2 (hereinafter referred to as 1/2, consistent with the nomenclature introduced by Gonzalez-Canche et al. [22]) with symmetrical lay-up: [C_0°_/C_0°_/PA/S/PA/C_0°_/C_0°_] (C-CFRP, S-steel, with rolling direction 90° to fiber direction, PA-polyamide 6 foil interlayer) resulting in a total thickness of about 1 mm. The metal layer was located in the core of the FML instead of the outer layers—unlike, e.g., GLARE—to address the challenge of electrical conductivity measurements of inner layers for future structural health monitoring or damage detection in materials testing. The constituents were dried overnight at 90 °C and subsequently consolidated at 260 °C and 25 bar for 20 min using a modified hydraulic upstroke press (SATIM, France, modified by Wickert, DE) with a shear edge tool. Tensile specimens were extracted from the FML sheets by water jet cutting along the 0° orientation of the fibers.

### 2.2. Specimen

**Figure 2:**
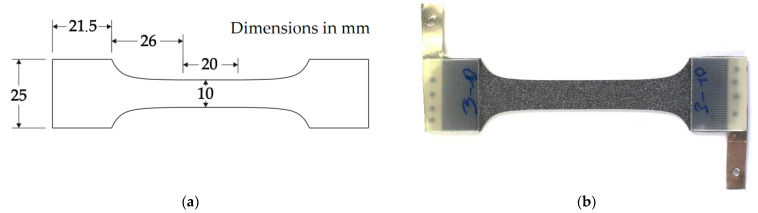
(**a**) Specimen geometry, (**b**) prepared specimen with adhesively bonded tabs, electrically conductive connection plates and speckle pattern for digital image correlation measurement.

For the investigations of this study, a dog bone-shaped specimen geometry was used. The geometry was self-developed with a spline as transition from the parallel gauge length to the clamping zone (Figure 2a), optimized for minimal stress concentration by finite element analysis. To further reduce stress concentrations at the clamping, glass fiber reinforced polymer tabs with a thickness of 1.5 mm were adhesively bonded to the clamping area of the specimen with epoxy glue (Figure 2b). A speckle pattern was spray painted onto the surface of the CFRP for digital image correlation (DIC) based strain measurement on the front surface of the specimen. For selected fatigue tests, the specimen were equipped with electrically conductive connection plates for measurement of direct current potential drop. These connection plates were placed in-between the protection tab and specimen and adhesively bonded to them. To ensure electrical conductivity between the steel and CFRP layers of the hybrid laminate and the connection plate, silver conductive paint was applied to the edges of the laminate.

### 2.3. Characterization and Testing Procedure

**Figure 3:**
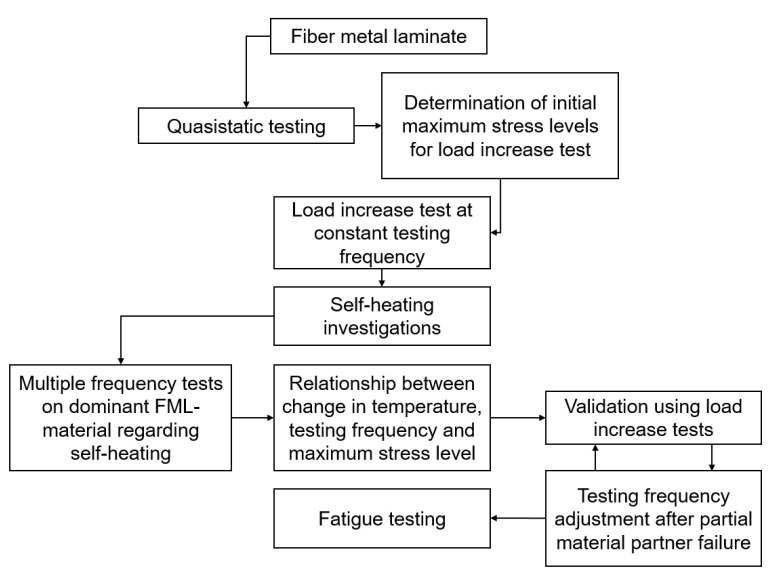
Flow chart showing the developed testing procedure for a reproducible characterization of the mechanical properties of fiber metal laminate under fatigue load.

The following flow chart (Figure 3) gives an impression of the developed testing procedure for a reproducible characterization of FML under tension loading. The used testing methods and connections between those are described more in-depth in the following Section 2.3.1 and Section 2.3.2. In general, this procedure is applicable to any material, but in case of the FML, which use multiple materials having different material properties, special consideration needs to be taken into account regarding the individual self-heating of each laminate partner.

Initially, quasistatic testing is carried out to determine tensile properties of the laminate and laminate material partners. These values can be used for material characterization, but also are needed to estimate maximum stress levels for load increase tests. Using the load increase test, which needs to be executed under a constant testing frequency, information about the extent of self-heating under fatigue load at different applied maximum stresses can be gathered. With that, multiple frequency tests are executed on the dominant FML material regarding self-heating. Using these tests, a relationship between the change in temperature, testing frequency, and maximum stress level can be established. The resulting testing frequencies for a selected maximum change in temperature are then validated using another load increase test to check if a further adjustment of testing frequencies other than given from the relationship are necessary due to partial material partner failure. Further adjustments can again be validated using load increase tests until a constant specimen temperature is achieved throughout the load increase test. This information can then be used to characterize the fatigue properties of the FML using a constant specimen temperature and therefore, excluding the influence of temperature change on the fatigue properties.

#### 2.3.1. Quasistatic Behavior

Tensile properties were identified in accordance with DIN EN 527-5 on a servo-hydraulic testing system (MTS 858 Mini Bionix II, F_max_ = ±25 kN, MTS Systems, Eden Prairie, MN, USA). The total strain was measured with the use of DIC. A minimum of three specimens were tested for single CFRP and steel layer material as well as the hybrid laminate.

#### 2.3.2. Cyclic Behavior

For fatigue characterization, load increase tests (LIT) and constant amplitude tests (CAT) were carried out on the same servo-hydraulic testing system as for the quasistatic tests. Fatigue load was applied with the use of a sinusoidal tension-tension load-time function at a stress ratio R = 0.1. LIT were conducted with a starting maximum stress σ_max,start_ = 50 MPa, followed by stepwise stress increase of Δσ_max_ = 25 MPa per ΔN = 2.5 × 10^3^ cycles up to specimen failure. For CAT, the maximum stress levels were derived from the LIT test results using the measurement information regarding points of characteristic microstructural changes to get an impression about the overall fatigue lifetime of the hybrid laminate.

To determine suitable fatigue testing frequencies, the laminate structure dependent self-heating was investigated with the use of multiple frequency tests (MFT) [15]. Since the CFRP is mostly influenced by self-heating, only this material out of the hybrid laminate was investigated. Therefore, the determined testing frequencies represent a conservative estimate for the hybrid laminate since the total self-heating is reduced due to the steel partner. Additionally, after a partial failure of the hybrid laminate (steel failure), the testing frequency can be adjusted to the remaining CFRP and effective stress. The MFT were conducted at maximum stresses of σ_max_ = 200 to 1000 MPa. The start frequency and change in frequency for each maximum stress level are shown in Table 1. Each frequency was held for 5 min during the MFT to enable temperature stabilization. Due to testing machine restrictions and required consistent high control accuracy, testing frequencies of 30 Hz were not exceeded. For a comparison to results at constant testing frequency, self-heating was also investigated in LIT at a constant testing frequency of f = 10 Hz. The finally determined frequencies for the CAT were chosen for a resulting, maximum increase in surface temperature of 2 K due to testing frequency during the test, resulting in a negligible impact of self-heating in the specimen’s cross-section on the mechanical properties while attaining acceptable testing duration compared to a constant testing frequency of f = 10 Hz.

**Figure 4:**
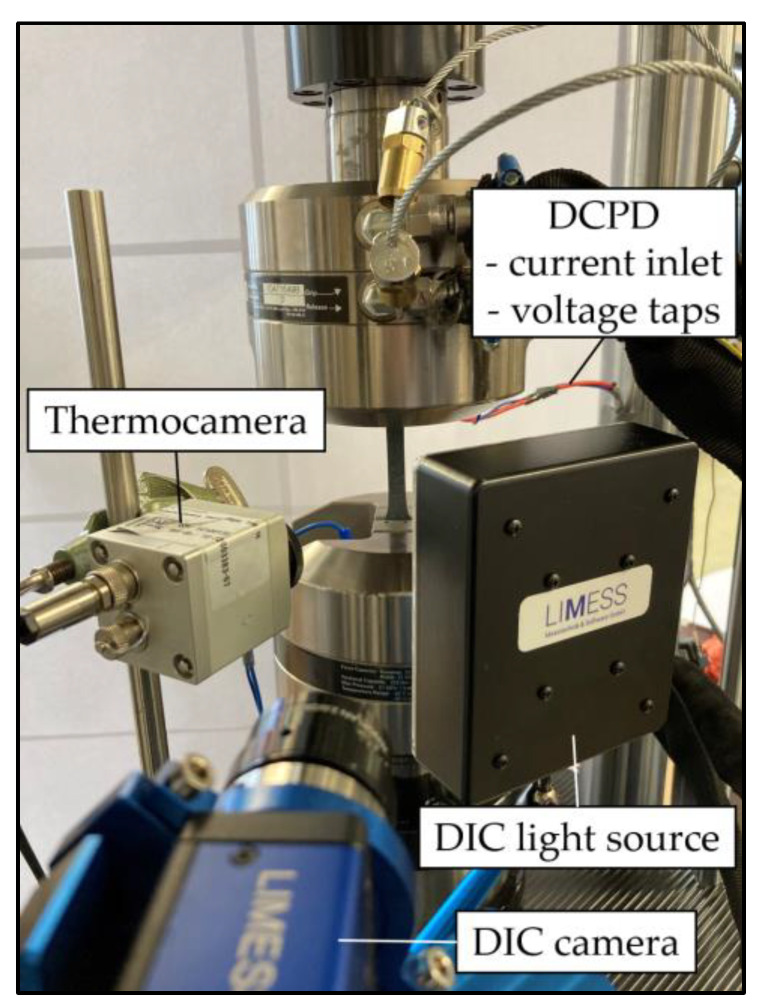
Experimental setup containing servo-hydraulic testing system and metrology instrumentation.

Figure 4 shows the experimental setup. Besides monitoring of the hybrid laminates’ change in dynamic stiffness C_dyn_ (change of stress divided through the change of piston displacement: (σ_max_ − σ_min_)/(s_max_ − s_min_)) the underlying microstructural changes were monitored by multiple sensors for correlation. A Limess Q400 DIC system (Limess Messtechnik und Software, Krefeld, Germany) containing one camera with a precision lens (28 mm focal length) was used to measure the 2-dimensional local deformations on the front specimen’s surface. Pictures were taken during the quasistatic tests time-triggered, while for the fatigue tests a Limess Maxtrigger box was used to acquire pictures at the maximum stress with an acquisition rate of every 100th cycle during LIT. This rate was adjusted for the CAT according to the used testing frequency, aiming at around 1000 pictures per test to achieve a sufficiently high data density while taking a processable amount of data volume into account. With the evaluation software Istra 4D V4.4.7 (Dantec Dynamics, Ulm, Germany) the total strain and maximum total strain were extracted from the DIC measurements using 25 mm virtual gauge line elements in the central section of the specimen.

The change in surface temperature was recorded using a MicroEpsilon TIM 160 (Micro-Epsilon Messtechnik, Ortenburg, Germany) thermocamera. Additionally, the change in electrical resistance of the hybrid laminate was measured during selected fatigue tests to indicate its potential for monitoring the electrical conductivity during load to conclude onto the damage state of the steel as well as CFRP. For this direct current potential drop measurement and Ohm’s law were applied. A Sorensen XG 100-8.5 power supply (AMETEK Programmable Power, San Diego, CA, USA) was used as an electric current source in combination with a National Instruments cDAQ 9174 and NI-9238 module (National Instruments, Austin, TX, USA) for voltage drop measurement.

**Figure 5:**
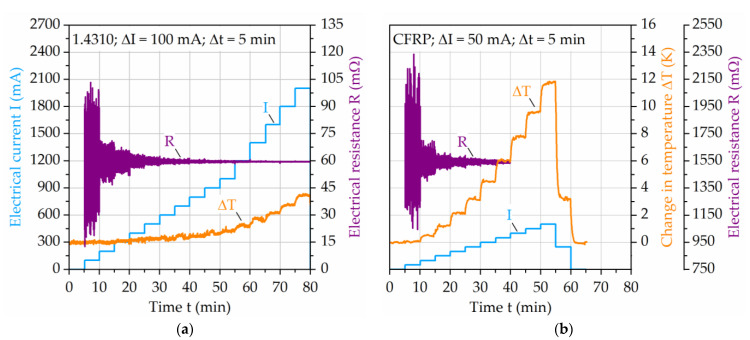
Influence of electrical current on the change in temperature of (**a**) 1.4310 and (**b**) carbon fiber reinforced PA6. The change in temperature axis in (**b**) is also valid for graph (**a**).

To ensure that the induced electric current does not increase the change in temperature of the specimen due to its electrical resistance, investigations were conducted regarding the temperature and electric current relationship of the laminate partners. The steel and CFRP have different electrical resistivity and temperature coefficients and therefore, lead to different magnitudes of resistance and temperature at applied electric current. Figure 5 shows the influence of electrical current on the change in temperature of a single layer of steel (Figure 5a) and CFRP (Figure 5b) of the hybrid laminate. The electrical resistance value scatter illustrates the measurement deviation for selected electric currents. While for steel up to an electrical current of 1.5 A the change in temperature stays below 2 K, for CFRP this is the case only up to 200 mA. After this, the temperature increases significantly with increasing electric current, which is why for the development of the testing procedure and fatigue performance characterization the maximum electric current of 200 mA is seen as a suitable value for the whole laminate to limit the temperature increase to a negligible level.

### 2.4. Thermal Residual Stress Calculation

Thermal residual stresses (TRS) occur due to the mismatch of the thermal expansion coefficients of the constituent materials. As a first estimation, the following Equation (1) can be used to calculate TRS (derived from [24]):

(1) $${\mathrm{TRS}}_{i}=E_{i}\cdot \Delta T\left({\mathrm{CTE}}_{\mathrm{FML}}-{\mathrm{CTE}}_{i}\right)$$

where index i denotes the constituent (CFRP, steel or PA interlayer), index FML the overall FML property, the temperature difference (ΔT) between consolidation and ambient temperature, and Young’s modulus (E_i_) of constituent i. CTE of FML can be calculated using the following Equation (2) [24]:

(2) $${\mathrm{CTE}}_{\mathrm{FML}}=\frac{E_{\mathrm{CFRP}}{\mathrm{CTE}}_{\mathrm{CFR}-\mathrm{PA}}V_{\mathrm{CFR}-\mathrm{PA}}+E_{\mathrm{steel}}{\mathrm{CTE}}_{\mathrm{steel}}V_{\mathrm{steel}}+E_{\mathrm{PA}}{\mathrm{CTE}}_{\mathrm{PA}}V_{\mathrm{PA}}}{E_{\mathrm{CFR}-\mathrm{PA}}V_{\mathrm{CFR}-\mathrm{PA}}+E_{\mathrm{steel}}V_{\mathrm{steel}}+E_{\mathrm{PA}}V_{\mathrm{PA}}}$$

V denotes the volume fraction of the corresponding constituent.

Table 2 summarizes the elastic material properties used as input taken from corresponding tests. Calculations were done for FMLs with and without PA interlayers.

For a detailed estimation of TRS, a finite element (FE) simulation was carried out using Abaqus 2020 (Dassault Systèms, Vélizy-Villacoublay, France). For the CFRP, an orthotropic material model was implemented. Engineering constants were taken from corresponding tests (E_1_ = 108 GPa, E_2_ = 6.95 GPa, ν_12_ = 0.3, G_12_ = 3.59 GPa, CTE_2_ = 101 ppm/K). For steel and PA plasticity was implemented using measurement data taken from tensile tests. The FML was modeled with shell elements and a composite layup. A homogeneous cooling of 240 K was carried out using a predefined field. Additionally, for one calculation the PA interlayers were replaced by E-glass twill weaves (E = 21.9 GPa, CTE = 23 ppm/K, thickness = 0.23 mm) reinforced PA to reduce the tensile TRS of the metal constituent.

## 3. Results and Discussion

### 3.1. Quasistatic Behavior

**Figure 6:**
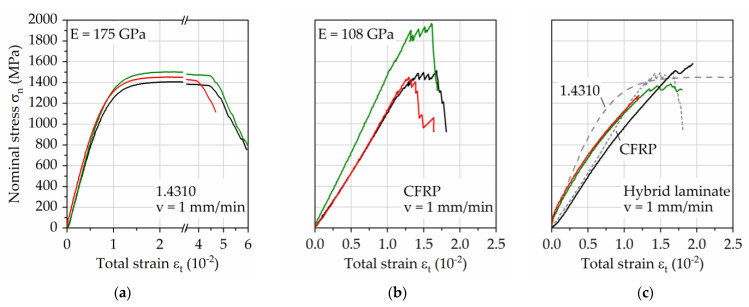
Stress–strain curves of (**a**) 1.4310, (**b**) carbon fiber reinforced PA 6, and (**c**) both combined as hybrid laminate in a 1/2-configuration. Each color represents one specimen tested. For the hybrid material the stress–strain curves of the constituents are given for comparison in the diagram.

The obtained results gained through quasistatic tensile tests are visualized in Figure 6 for the single materials (Figure 6a) steel 1.4310 and CFRP (Figure 6b) as well as for the hybrid laminate (Figure 6c). Up to the nominal stress level of around 1200 MPa, before the appearance of a significant increase in plastic strain of the steel, the stress–strain development of steel and the CFRP show similar trends. While the strain of the steel increases constantly after this point due to plasticity until failure, CFRP is able to withstand further load with a linear increase in strain as well as partial fiber breakage. The combination of both materials, therefore, should represent a good match regarding quasistatic properties, which is visible in Figure 6c. The resulting stress–strain curves can be seen as a representative overlay of the single materials stress–strain curves, besides the missing visibility of plasticity of the steel due to the dominant CFRP stiffness. In its hybrid laminate state, this combination retains the high mechanical performance of steel and CFRP, while improving the electrical conductivity of CFRP.

### 3.2. Testing Frequency Induced Self-Heating of the Hybrid Laminate

**Figure 7:**
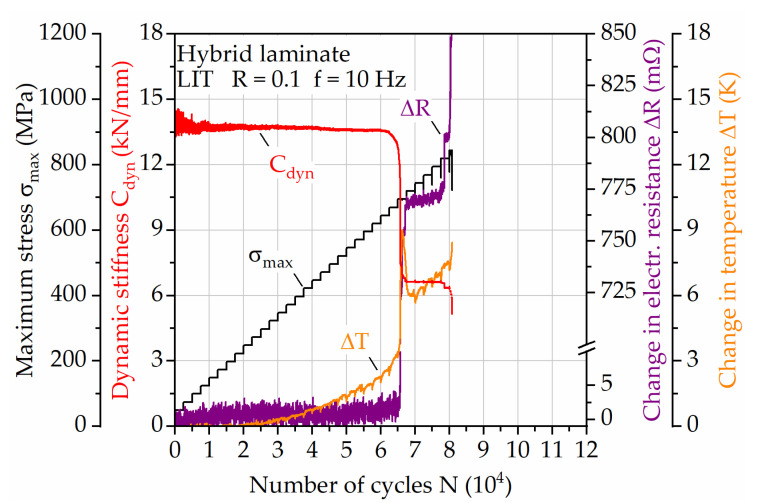
Load increase test for hybrid laminate in 1/2-configuration of 1.4310 and carbon fiber reinforced PA 6; development of dynamic stiffness, change in temperature and electrical resistance when testing with constant frequency.

In a first instance, a LIT with a constant testing frequency of 10 Hz was conducted on the hybrid laminate to get an impression regarding the self-heating induced change in temperature of the specimen (Figure 7). With increasing maximum stress and therefore strain, the surface temperature increases up to around 4 K until failure of the steel layer (at about 66,000 cycles). The initiation of upcoming steel layer failure is visible in the dynamic stiffness and electrical resistance values, showing that the measurement of the latter can be used to replace conventional measurement techniques for damage state assessment. In the following, where the load is applied just to the remaining CFRP-layers, the electrical resistance increases with failing fibers and the temperature increases up to 8 K before failure. The damage induced change in temperature can be a relevant factor that needs to be taken into account for, e.g., the upcoming steel failure, but is considered negligible at times where the mechanical properties show constant development. Looking at the results a significant temperature increase is visible when using a constant testing frequency of 10 Hz.

**Figure 8:**
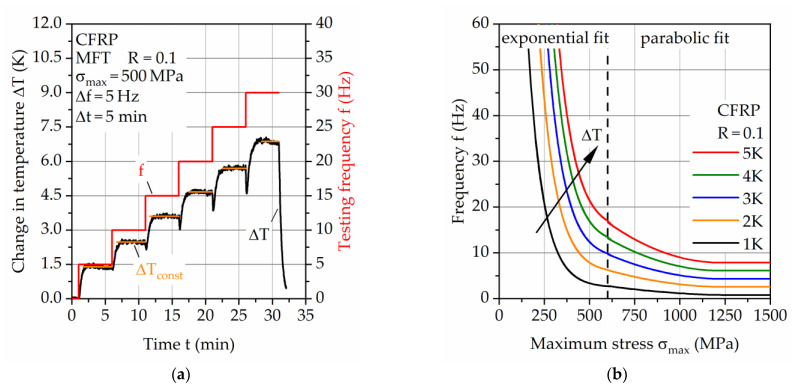
(**a**) Exemplary multiple frequency test on carbon fiber reinforced PA6 and (**b**) derived testing frequency-maximum stress relationship visualized for ΔT from 1 to 5 K.

Figure 8a shows exemplarily the frequency dependent change in temperature measured during MFT at σ_max_ = 500 MPa. The testing method enables the measurement of stable temperature plateaus during fatigue load for a characterization of the temperature-testing frequency relationship of CFRP. For this test, the change in temperature appears as constant increases with establishment of temperature plateaus up until the maximum testing frequency of f = 30 Hz. If in such a test continuous increases in temperature occur within one frequency step, which hints at are more complex causes such as too excessive strain rates and material damage, these steps are not considered for the determination of the temperature and testing frequency relationship. Figure 8b visualizes the derived testing frequency-maximum stress relationship for ΔT from 1 to 5 K. Due to different temperature development before and after σ_max_ = 600 MPa, two different fitting functions are needed and implemented, in this case, an exponential and parabolic fit.

**Figure 9:**
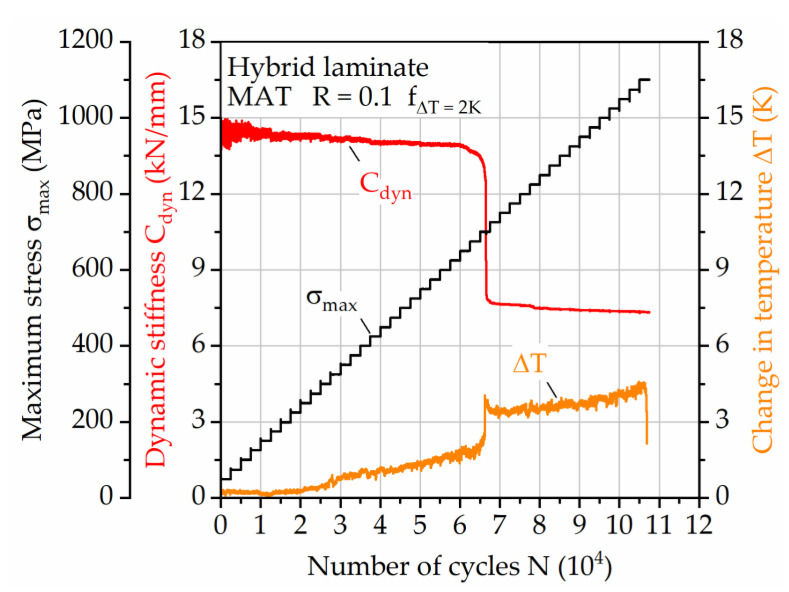
Load increase test for hybrid laminate in 1/2-configuration of 1.4310 and carbon fiber reinforced PA 6, showing the development of dynamic stiffness and change in temperature when testing with adjusted frequency for a change in temperature of 2 K.

When applying the testing frequency relationship from Figure 8b, considering a maximum change in surface temperature of 2 K, to a LIT on the hybrid laminate, the results below (Figure 9) are achieved. The change in temperature develops until σ_max_ = 400 MPa similarly compared to the LIT with a constant testing frequency of 10 Hz, since only after this step the constant testing frequency of 10 Hz is higher than the adjusted ones. After that, the change in temperature does not reach 2 K until failure of the steel layer, since the determined frequency values are valid for the CFRP layer, rather than the whole hybrid laminate. After the failure of the steel layer the change in temperature increases above 2 K. This is related to the significantly higher effective stress on the CFRP layers, leading to highly increased strain and therefore self-heating.

**Figure 10:**
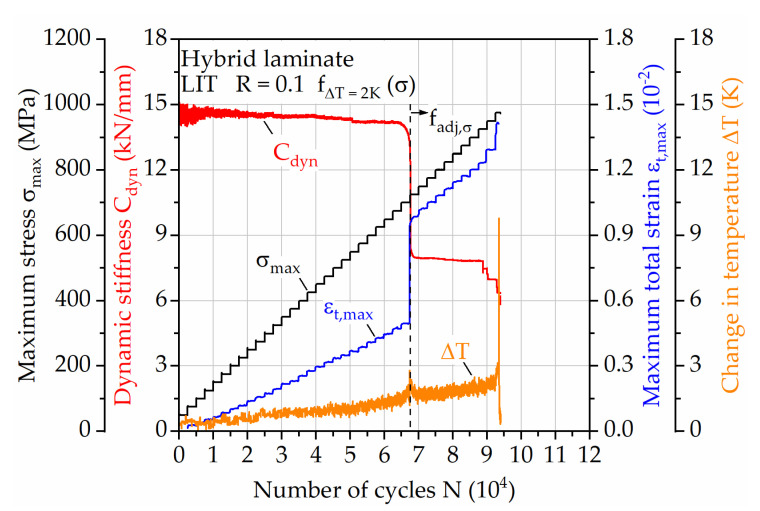
Load increase test for hybrid laminate in 1/2-configuration of 1.4310 and carbon fiber reinforced PA 6, showing the development of dynamic stiffness, change in temperature and maximum total strain for testing with adjusted frequency for a change in temperature of 2 K in combination with further adjustment of the frequency after failure of 1.4310 related to the effective stress.

To compensate for this temperature increase, a laminate integrity dependent adjustment of the testing frequencies needs to be taken into account. When considering the remaining cross-sectional area of the specimen after the failure of the steel, presuming a mostly intact CFRP laminate, the testing frequencies can be adapted to the effective stress on the CFRP layers. Using this approach, the maximum change in surface temperature does not exceed 2 K during LIT until specimen failure (Figure 10). The presumption of mostly intact CFRP seems valid, since the change in temperature, using the determined frequencies for CFRP (Figure 8b), is kept as low as it is supposed by the determined relationship. The DIC-based measured maximum total strain on the specimen’s surface supports this. Immediately after the failure of steel the related stiffness drop correlates to a change in maximum total strain, leading to different slopes of the linear increase in strain from start until failure of the hybrid laminate. Only at the end of the test, significant changes in stiffness, strain, and temperature are visible, leading to the non-linear change in surface temperature, which is material damage related and therefore not covered by the used method of testing frequency adjustment.

### 3.3. Fatigue Behavior

**Figure 11:**
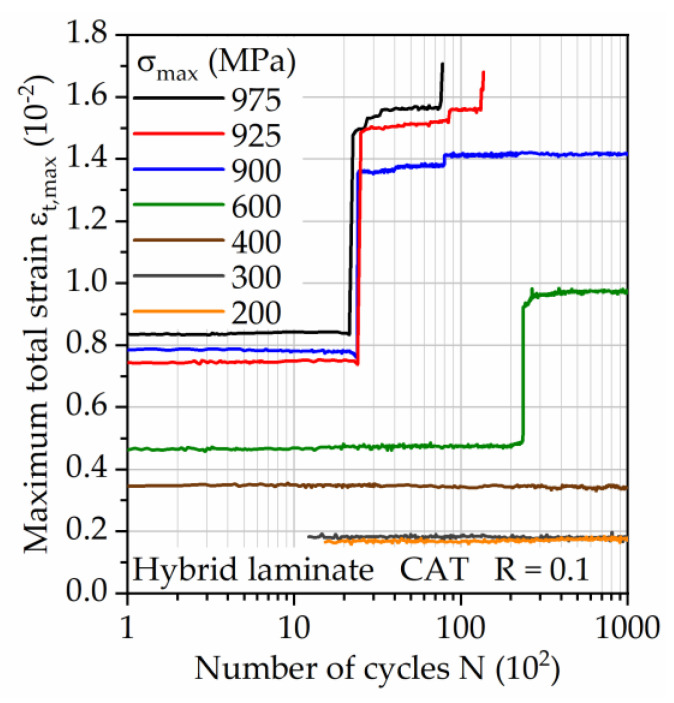
Maximum total strain measured via DIC during CAT on the surface of the hybrid laminate in 1/2-configuration of 1.4310 and carbon fiber reinforced PA 6.

As shown in Section 3.4 in the LIT results, the hybrid laminate exhibits a stepwise damage sequence and thus undergoes graduated damage development. The failure of the steel layer is the most prominent, leading to a significant change in the stiffness of the laminate. The DIC measured maximum total strain ε_t,max_ (Figure 11) shows that with increasing maximum stress an earlier failure of the steel layer is evident, represented by the high sudden increase in strain. This failure mode highlights that the steel layer is the weakest part of the laminate. Considering the residual stress calculations, which showed to be in tensile direction and therefore, reduce the lifetime for the investigated stress ratio of 0.1, the significance of the influence of thermal residual stresses superimposed to external stresses is an important factor.

After the failure of the steel layer, the CFRP endures higher stress proportional to the loss in cross sectional area of the steel layer. This leads at maximum stresses higher than 600 MPa to initial partial fiber detachment and failure at the edges and specimen radii, visible in the sudden and afterward steady increase in strain after steel failure. This propagates at stresses higher than 900 MPa and ultimately reduces lifetime significantly.

**Figure 12:**
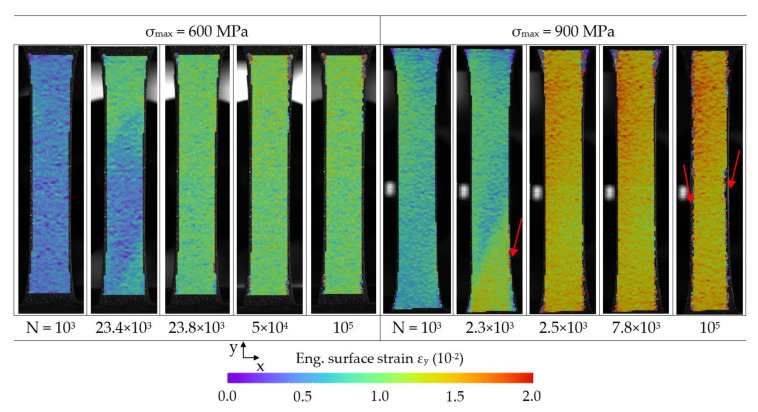
DIC strain measurement during CAT with σ_max_ of 600 and 900 MPa on the surface of the hybrid laminate in 1/2-configuration of 1.4310 and carbon fiber reinforced PA 6. Selected cycles are shown, visualizing the strain development before and after 1.4310 failure as well as fiber failure at the edges of the laminate (red arrows).

Figure 12 shows the local strain occurring on the CFRP surface during CAT with σ_max_ of 600 and 900 MPa, which highlights the failure of the steel layer and strain development. The observable steel failure, which initiation is visible by the local increases in strain at 23.4 × 10^3^ cycles for 600 MPa and at 2.3 × 10^3^ cycles for 900 MPa, shows to be comparable despite the different applied maximum stress. The reason for the angled strain pattern during steel failure lies in the one-sided crack initiation, leading to strain accumulation development in the adjacent region. When steel failure starts, in only a few hundred cycles the whole CFRP layers delaminate, leading to nearly a doubling in strain. The damage mechanisms and resulting change in CFRP load are the same, leaving only the initiation time as a major difference. After the steel failure, the increase in strain for σ_max_ = 900 MPa leads to the continuous development of fiber detachment and failure visible at the edges of the laminate (*n* = 2.5 × 10^3^ to 10^5^), which still does not lead to failure, demonstrating the high load bearing capabilities at even higher effective stresses considering the missing cross-sectional area of the steel layer.

**Figure 13:**
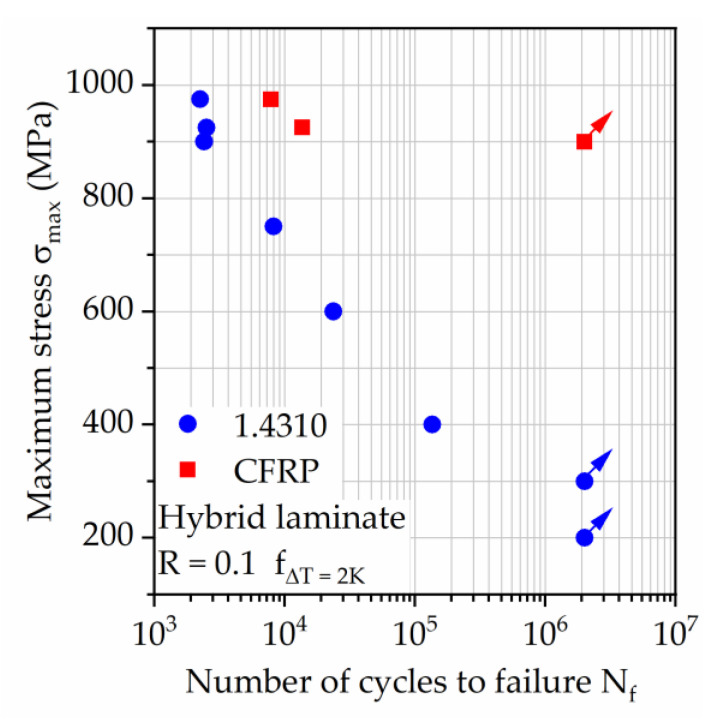
S–N relationship for hybrid laminate in 1/2-configuration of 1.4310 and carbon fiber reinforced PA 6. Failure for 1.4310 and postponed failure of carbon fiber reinforced PA 6 are marked. For tests with σ_max_ below 900 MPa the tests were stopped after steel failure, since carbon fiber reinforced PA 6 proved to withstand 2 × 10^6^ cycles at 900 MPa after failure of 1.4310.

As a result, the hybrid laminate can withstand significant structural damage without catastrophic failure while still delivering high performance during tension-tension loading. Looking at Figure 13, the S–N relationship shows that only at comparably low maximum stresses of 300 MPa or lower the steel layer remains intact, maintaining the integrity of the hybrid laminate for the full lifetime of 2 × 10^6^ cycles investigated in this study. This aspect has to be kept in mind when considering such a hybrid laminate for applications, where the electric conductivity of the structure is a necessary property, e.g., for electrical data transfer to substitute, e.g., electrical cables. The relationship between applied maximum stress and number of cycles to failure of the steel layer (Figure 13, blue dots) follows almost a straight line in the half-logarithmic graph and can therefore be described as a power function, which is well suited for lifetime prediction using only a few tests. Minimizing the residual stresses occurring in the steel layer should lead to longer retention of the steel layers load bearing capabilities and thus, longer lifetime. This possibly is simply describable with help of the S–N graph through a shift of the steel layer lifetime to the right, depending on the intensity of residual stress reduction.

### 3.4. Residual Stresses

**Figure 14:**
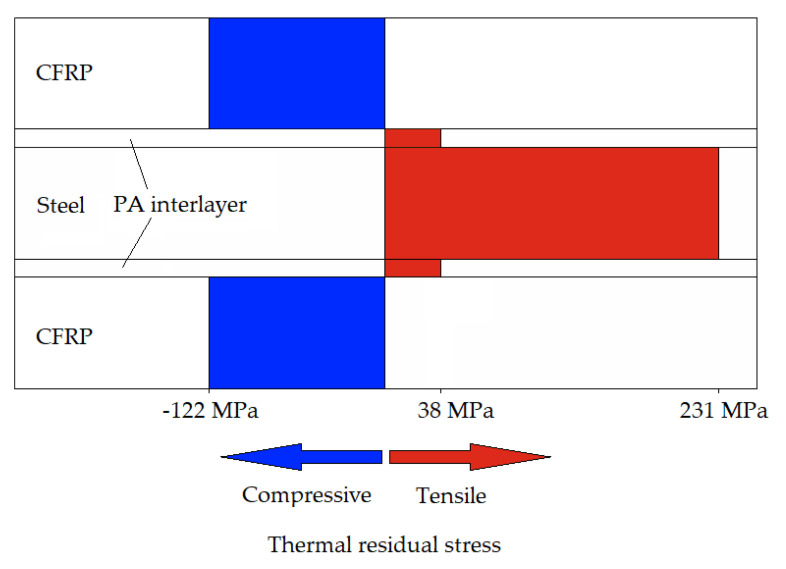
Exemplary visualization of the calculated thermal residual stress (numerical simulation with orthotropic material model) in the investigated hybrid laminate.

Stresses calculated analytically and numerically using the properties and calculation methods presented in Chapter 2 are summarized in Table 3. Figure 14 visualizes an exemplary TRS constellation.

Generally, it has to be noted that all the calculated stresses have to be considered as an idealized upper limit. For all calculating methods temperature dependent properties and viscous material behavior were neglected. Especially at elevated temperatures, it can be expected that residual stresses are partly reduced due to relaxation. Furthermore, a rigid connection of both components at the interface will not occur while the polymer is in a liquid state, so the effective temperature difference can be expected to be less than 240 K.

The different calculating methods (analytical and numerical) yield very different results. Stresses calculated analytically are about 25% higher than stresses calculated numerically with an orthotropic material model. In comparison, the lower TRS from the numerical method can be considered the most accurate since anisotropy and transversal strains are neglected by the other simplified method. However, due to the described calculating difficulties of all methods, TRS need to be accurately measured for a more precise stress analysis.

The PA interlayer only shows a minor influence on TRS. The compressive stresses in CFRP are intensified by about 2% and the tensile stresses in steel are reduced by about 1–3%. This minor influence is due to the low modulus and thickness fraction of PA despite its high CTE.

The calculation using glass weave reinforced PA as interlayer shows the lowest tensile TRS in the metal constituent and higher compressive TRS in the CFRP. This is due to the higher CTE of the FML, resulting from the relatively high CTE and moderate stiffness of the glass weave reinforced PA interlayer. The higher CTE of the FML causes a larger shrinkage during cooling, yielding in the different TRS distribution.

Given the tensile nature of the residual stresses in the metal constituent, a detrimental influence on fatigue strength is evident [13]. The results of the quasistatic investigations shown in Figure 6 show how the performance of the overall laminate can be affected by TRS since the combination leads to lower ultimate tensile strength compared to the single materials. Therefore, investigating and reducing TRS are critical parts of fatigue/lifetime analysis.

## 4. Conclusions and Outlook

The following conclusions can be drawn from the investigations on the hybrid laminate in 1/2-configuration of 1.4310 and carbon fiber reinforced PA 6:

For a comparative characterization of mechanical properties, a testing procedure was designed and evaluated, which considers prevention of the mostly polymer related self-heating effect, as well as the laminate integrity dependent changes in this regard due to increases in effective stress after the failure of the metal component. Using multiple frequency tests, a relationship between the self-heating and testing frequency was found and used to enable fatigue tests with changes in temperature up to a defined value while retaining as short as possible testing durations. Using the load increase tests, it was shown that an additional frequency reduction after the failure of the steel layer is necessary to maintain the self-heating of the remaining hybrid laminate, excluding temperature as a possible influence regarding the fatigue capability.

The hybrid laminate proved to be capable of high performance, showing a fatigue lifetime of a minimum of 2 million cycles at a maximum stress of 900 MPa for a stress-ratio of 0.1. The steel layer, which is the weaker material in the hybrid laminate, is failing first, which needs to be taken into account when using this laminate also because of its good electric conductivity. A measurement of the laminates’ electrical resistance revealed that the state of electrical conductivity can be monitored reliably throughout damage processes, but when considering long-term measurement in industrial applications other factors like electric current induced acceleration of corrosion processes need to be kept in mind and evaluated profoundly.

Due to the differences in coefficients of thermal expansion of 1.4310 and CFRP, the hybrid laminate leads to thermal residual stresses, which impact the mechanical properties. The used calculation method gives an impression about the quantitative extent of the occurring thermal residual stresses for each layer contained in the hybrid laminate. The CFRP component underlies compressive residual stresses around 120 MPa, while the steel exhibits tensile residual stresses around 230 MPa. For a closer consideration of thermal residual stresses, precise measurements need to be included in a stress analysis. Many parameters such as temperature dependent viscoelasticity or effective temperature difference are very difficult to incorporate into a bare calculation without validation. Possible measurement methods include X-ray diffraction, modified hole drilling, or the observation of yield strength shifting.

Considering tension-tension loading, the occurring thermal residual stresses contribute negatively. To enhance lifetime performance, a reduction of the tensile thermal residual stresses of the steel layer needs to be approached in future work. Exploiting the metals’ plasticity, post-stretching of the hybrid laminate decreases thermal residual stresses or even induces compressive stresses in the steel layer. In addition, adjusting the FML layup using different interlayer materials can have a beneficial effect due to different stiffness distributions and the resulting coefficients of thermal expansion of the hybrid laminate, as discussed in the calculation using a glass weave PA interlayer.

Further, the aspect of corrosion needs to be investigated in detail for such laminate configurations, since galvanic corrosion is known to occur between steel and carbon fiber in a large extent and the assessment of remaining service life is important. Therefore, continuous monitoring of electrical conductivity during salt-spray-tests as well as within combined in situ corrosion fatigue tests could lead to necessary data for determining advanced lifetime prediction methods.

## Figures and Tables

**Table 1:** Parameters for multiple frequency tests for laminate dependent self-heating investigation.

σ_max_ (MPa)	200	300	400	500	600	700	800	900	1000
f_start_ (Hz)	5	5	5	5	2	2	2	2	2
Δf (Hz)	5	5	5	5	2	2	2	2	2

**Table 2:** Material properties for stress calculation.

Material	E (GPa)	CTE (ppm/K)
CFRP (0°)	108	2.85
Steel	175	16.5
PA interlayer	1.844	95

**Table 3:** Calculated thermal residual stresses (S11 → fiber direction) using different methods and composite layups.

Interlayer	Calculation Method	Material Model	CFRP	Steel	Interlayer
			(MPa)		
none	analytical	-	−158	317	-
none	numerical	orthotropic	−119	239	-
50 µm PA	analytical	-	−162	311	38
50 µm PA	numerical	orthotropic	−122	231	38
Glass weave/PA	numerical	orthotropic	−148	191	69

## Data Availability

Not applicable.
